# Applying the revised Chinese Job Content Questionnaire to assess psychosocial work conditions among Taiwan's hospital workers

**DOI:** 10.1186/1471-2458-11-478

**Published:** 2011-06-18

**Authors:** Tsair-Wei Chien, Wen-Pin Lai, Hsien-Yi Wang, Sen-Yen Hsu, Roberto Vasquez Castillo, How-Ran Guo, Shih-Chung Chen, Shih-Bin Su

**Affiliations:** 1 Department of Management, Chi-Mei Medical Center, Taiwan; 2 Department of Hospital and Health Care Administration, Chia-Nan University of Pharmacy and Science, Tainan, Taiwan; 3 Department of Emergency Medicine, Chi-Mei Medical Center, Taiwan; 4 Department of Nephrology, Chi-Mei Medical Center, Taiwan; 5 Department of Psychiatry, Chi-Mei Hospital, Liouying, Taiwan; 6 Director of Local Integral Health Assistance System (SILAIS), Carazo, Nicaragua, Central America; 7 Department of Environmental and Occupational Health, National Cheng Kung University, Taiwan; 8 Institute of Biomedical Engineering, Southern Taiwan University, Tainan, Taiwan; 9 Department of Biotechnology, Southern Taiwan University, Tainan, Taiwan; 10 Department of Family Medicine, Chi-Mei Medical Center, Tainan, Taiwan

## Abstract

**Background:**

For hospital accreditation and health promotion reasons, we examined whether the 22-item Job Content Questionnaire (JCQ) could be applied to evaluate job strain of individual hospital employees and to determine the number of factors extracted from JCQ. Additionally, we developed an Excel module of self-evaluation diagnostic system for consultation with experts.

**Methods:**

To develop an Excel-based self-evaluation diagnostic system for consultation to experts to make job strain assessment easier and quicker than ever, Rasch rating scale model was used to analyze data from 1,644 hospital employees who enrolled in 2008 for a job strain survey. We determined whether the 22-item Job Content Questionnaire (JCQ) could evaluate job strain of individual employees in work sites. The respective item responding to specific groups' occupational hazards causing job stress was investigated by using skewness coefficient with its 95% CI through item-by-item analyses.

**Results:**

Each of those 22 items on the questionnaire was examined to have five factors. The prevalence rate of Chinese hospital workers with high job strain was 16.5%.

**Conclusions:**

Graphical representations of four quadrants, item-by-item bar chart plots and skewness 95% CI comparison generated in Excel can help employers and consultants of an organization focusing on a small number of key areas of concern for each worker in job strain.

## Background

With the increasing concern about job strain, many researchers have addressed psychosocial job strain and its adverse effects on health [1-3]. The topic of occupational strain receives considerable research attention and is an important occupational safety and health issue. Research evidence shows that perceived stress overtime and quantitative job overload negatively impact worker mental health. The prevalence of workers reporting mental stress due to job overload increased from 51% in 1982 to 63% in 1997 among Japanese workers and job stressors causing 60% to 80% of occupational accidents and absenteeism in Taiwan [2,4].

### Job Content Questionnaire (JCQ)

The Job Content Questionnaire (JCQ) based on Karasek's Demand-Control Model, i.e., subscales of job control and psychological demands, is the most popular[1,5], among many instruments designed for the assessment of psychosocial work environment. Karasek and Theorell later incorporated this questionnaire with work-related social support (i.e., subscales of supervisor support and co-worker support) as the Demand-Control-Support Model [1]. Most of the epidemiological studies utilize the JCQ to measure the 'content' of a respondent's work tasks in a general manner, which is applicable to all jobs and workers for predicting job related stress and coronary heart disease in many countries[6-11], and also they are used for studying worker motivation, job satisfaction, absenteeism, and labor turnover [5,8]. The 22-item core JCQ Chinese version (C-JCL) was verified and published in an international journal [12]. However, few papers illustrated a graphical presentation of job strain interpretation for workers, especially in friendly used Excel, to help mental health experts focussing on a small number of key areas of concern.

### Four exposure groups classified by JCQ

Job Demand-Control (or Job Strain) Model involves four quadrants displaying psychological job demands (PD), namely high and low strain and active and passive jobs [13,14], those are jointly separated by another subscale of decision latitude (DL). High strain jobs with low DL and high PD are particularly perceived to cause the most adverse psychological reactions [15]. Many prospective studies have supported this hypothesis. Four exposure groups including (i) high strain (exposed to low DL and high PD); (ii) low strain (high DL and low PD); (iii) passive (low DL and low PD); and (iv) active (high DL and high PD) could be graphically illustrated for identifying examinee's high or low job strain.

### Study Objectives

Calculating a sum of weighted item scores for each subscale of those 22 items by the specific calculation formulas of the originators is subjective and cumbersome [1,12]. None study, until now, was conducted using the 22-item C-JCL to generate diagnostic reports for workers by a graphical representation to identify their job stresses.

For the graphical representations, it is worthy of (1) depicting a four-quadrant diagram reporting individual job stressors and strains; (2) developing item-by-item bar chart plots to examine person's response on items which can help consultants focusing on a small number of key areas of concern; and (3) make a comparison of the inter-group and inter-occupation differences by item-by-item box plots of disclosure for a sample using skewness coefficients and 95% confidence intervals (CIs) to justify occupational job stress whether it is positive or negative skew to an issue (i.e., item or question).

## Methods

### Study Participants

The setting was a 1,200-bed hospital located in southern Taiwan in which 2,791 workers participated in a job perception examination in August of 2008. Self-administered questionnaires were distributed to all the employees of the hospital, and 1,863 employees responded (return rate, 66.75%). We further excluded 219 participants who had incomplete information on age, gender, and those with work tenure of three months or less, and part-time workers. As a result, 1,644 workers (or 58.9% of 2,791) were available for this study (Table 1).

**Table 1:** Demographic Characteristics (N = 1,644)

	Male	Female	Total
	n = 290	n = 1354	n = 1,644
Age (Avg. yrs)	38	33	35
Age (%)			
21~30	25%	43%	40%
31~40	45%	39%	40%
41~50	20%	13%	15%
51~60	7%	2%	3%
Work tenure			
Within 1 yr	25%	8%	12%
1-5	6%	39%	33%
5-10	24%	17%	18%
Above 10 yrs	41%	36%	37%
Job title			
Physician	35%	4%	10%
Nurse	18%	64%	56%
Technician	22%	16%	17%
Administrator	24%	15%	17%
Others	1%	1%	1%

### Steps of instrument selection

#### 1. Questionnaire

With permission from the author of the Chinese Version of the JCQ (C-JCL) [12], 22 core items were selected, including the four subscales such as DL measured by nine items, i.e., skill job discretion (6 items) and decision authority (3 items), PD measured by five items, and supervisor and co-worker support, both measured by four items. For each item, the response was recorded using a four-point Likert scale ranging from 1 (strongly disagree) to 4 (strongly agree). We used the WINSTEPS software [16] to estimate each person's psychometric characteristics by combining all 22 items and separating items for respective subscales.

#### 2. Validation Procedure

##### (1). Rasch modeling analysis

We programmed an Excel module helping workers examine their job strain. The 22 core items are required to identify number of factors extracting from data. Rasch [17] model was applied to analyze data in Excel for the assessment of job strain for workers. The great advantage of using a Rasch model is that it is an explicit empirical model of the latent trait, which allows for the testing of data fit to the model while dealing with missing response values and transforming raw scores into a linear interval score in a logit (log odd) unit [18-21].

##### (2) Dimensional checking for C-JCQ

We detected the number of factors for C-JCQ using parallel analysis [22], one of the most recommended methods for dealing with the number-of-factors-to-retain problem [23,24] to observe C-JCQ dimensionality. ViSta version 7.9.2.6 (2010, April) [25] was performed to plot the graphical parallel analysis with 95% confidence intervals.

### Prevalence rate of high strain in hospital workers

We used formula (1) to calculate the prevalence rate to uncover participants with work-related stress compared to the total population at risk of contracting that stress.(1)

### Excel module combined with WINSTEPS estimating measures

Rasch transformed scores in each domain will be generated by separately estimating person measures using the Excel module which was incorporated with WINSTEPS software to easily estimate the individual measures. The diagram of four quadrants in Excel were plotted to classify workers by DL and SD subscales for individual examinees. Item-by-item bar chart plots to examine person's placement will be developed in Excel. Those two graphical representations are expected to help consultants to easily, quickly and clearly identify job strain for each worker.

### Job stress monitored by skewness analyses

Skewness coefficient was suggested to report in evaluating dissimilarity of examinee group [26]. In a skewed (unbalanced, lopsided) distribution, the mean is farther out in the long tail than is the median. If there is no skewness (i.e., the distribution is symmetric) then the mean = the median = the mode. It can be applied to workplace for graphical health performance reports as an indicator of examinee group discrimination on a single item regarding job stress.

If we are concerned with the occupational (or demographic) group which is ascribed to the job stress with positively skewed population which indicates that most of them perform worse (i.e., most persons with lower responding score) on the single item. It is required using computer efficiently to screen them for further assessment. The skewness coefficient and its 95% CI yielded by Bootstrap [27] for all types of occupational group on items are estimated by author programmed Excel-VBA module.

### Ethics Review Board Approval

The protocol of this study was approved by the Research and Ethics Review Board of Chi-Mei Medical Center. All authors certify that there are no known conflicts of interest with any third party.

## Results

### Descriptive Statistics

Table 1 summarizes the demographic characteristics of the study population. The average age of the men and women were 38 and 33 years, respectively, and the mean duration of work tenure was about 4.8 years for both men and women. The majority of respondents were nurses (56%), whereas only 10% were physicians.

### Checking dimensions of C-JCQ

All the 22 items fit the expectations for the Rasch model fairly well, with infit and outfit MNSQs between 0.60 and 1.40, respectively (Table 2). The most difficult (i.e., the least occurrence) item to appear was conflicting work (item 14; 2.76 logits; *SE *= 0.04). The easiest (i.e., the most frequent) to present was learning new things (item 1; -2.18 logits; *SE *= 0.05). Cronbach's alpha coefficient is 0.8 similar to the previous study (Cheng, Luh, Guo, 2003) [12].

**Table 2:** Item difficulties and unidimensional test by MNSQ within 0.60 and 1.40

	Strongly disagree, Disagree, Agree, Strongly agree	Difficulty	**S.E**.	Infit	Outfit
SD	01.My job requires that I learn new things	-2.18	0.05	1.00	1.01
	02.*My job involves a lot of repetitive work	2.42	0.04	1.01	1.11
	03.My job require me it be creative	-0.86	0.05	0.96	0.94
	05.My job requires a high level of skill	-0.76	0.05	1.25	1.23
	07.I get to do a variety of different things on my job	-0.82	0.05	1.19	1.17
	09.I have an opportunity to develop my own special ability	0.25	0.04	0.92	0.93

DA	04.My job allows me to make a lot of decision on by job	0.02	0.04	1.00	1.00
	06.*On my job, I have very little freedom to decide how I do my work	0.41	0.04	1.07	1.10
	08.I have a lot of influence about what happens on my job	0.48	0.04	0.90	0.92

PD	10.* My job require working very fast	2.18	0.04	1.29	1.38
	11.*My job requires working very hard	2.46	0.04	1.25	1.33
	12.I am not asked to do an excessive amount of work	1.44	0.04	1.34	1.38
	13.I have enough time to get the job done	0.55	0.04	1.27	1.29
	14.I am free of conflicting demands that others make	2.76	0.04	1.10	1.23

SS	15.My supervisor is concerned about the welfare of those under them	-0.5	0.04	0.98	0.95
	16.My supervisor pays attention to what I am saying	-0.62	0.04	0.76	0.73
	17.My supervisor is helpful in getting the job done	-0.83	0.05	0.76	0.73
	18.My supervisor is successful in getting people to work together	-0.76	0.05	0.78	0.74
CS	19.People I work with are competent in doing their jobs	-1.14	0.05	0.77	0.74
	20.People I work with take a personal interest in me	-1.42	0.05	0.69	0.68
	21.People I work with are friendly	-1.52	0.05	0.74	0.72
	22.When needed, my colleagues will help me?	-1.54	0.05	0.74	0.73

Note. * reverse scoring response

SD: skill discretion, DA: decision authority, PD: psychological job demand, SS: supervisor support, CS: coworker support

**Figure 1:**
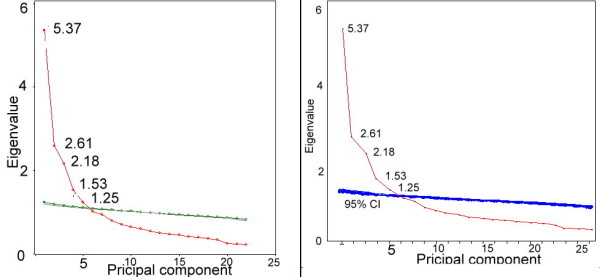
**Parallel analysis for C-JCQ items**. Note. Five factors were extracted by 95% CI of parallel analysis in the left panel.

Tennant and Pallant suggested [28] that exploratory factor analysis (EFA), especially using parallel analysis (PA)[22], should be undertaken to ensure the dimensionality of study data before conducting a Rasch analysis. Parallel analysis was performed to identify that C-JCQ has five factors extracted from those 22 items (Figure 1).

### Excel module plotting graphical representations of four exposure groups

**Figure 2:**
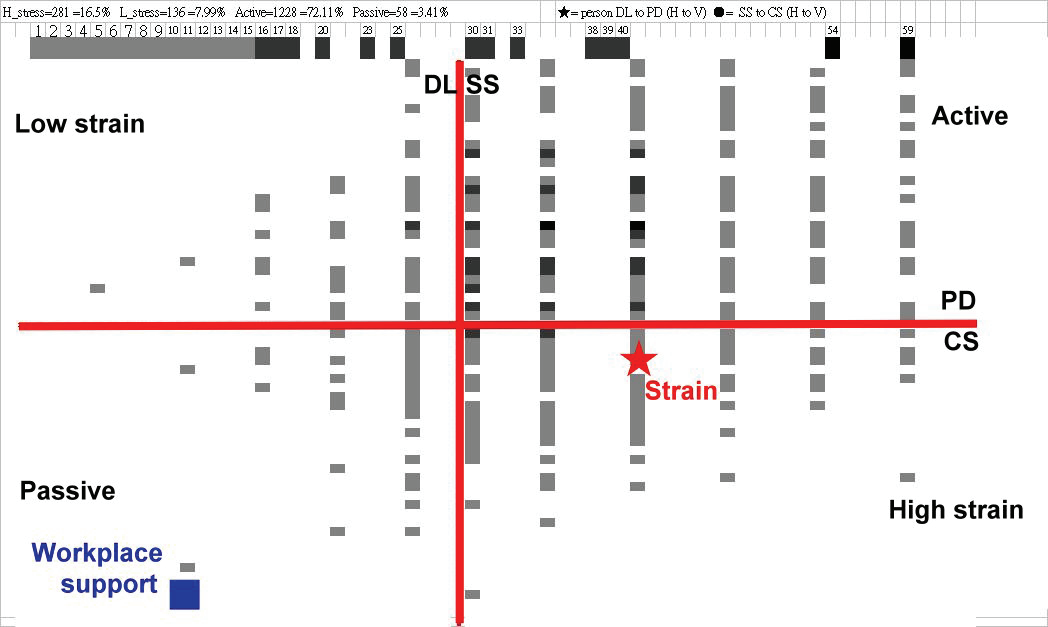
**JCQ report in Excel showing high strain with low workplace support for an examinee**. Note. The darker the color, the greater the number.

In order to develop a self-administered assessment to help workers diagnose their job strain, we illustrate a worker corresponding to the relationship between four exposure groups in Figure 2. They were 281 (16.5%), 136 (7.99%), 1228 (72.11%) and 58 (3.41%) for high and low strain, and active and passive jobs, respectively. In the diagram, we see that the number that occurred in each group is shown in different color (the darker the color, the greater the number). The worker's scores displayed in the diagram showing high strain in the VI quadrant (symbolized by an asterisk) and low scores of SSs (supervisor support) and CSs (co-worker support) in the III quadrant (symbolized by a square), indicating that the worker should be paid more concern for an obviously depressive disorder on job environment without satisfying social support in the workplace. The graphical representation in Excel can help consultants examine the relation of worker's job strain and support from supervisor and co-worker so that they can be easily, quickly and clearly identified.

### Excel module plotting item-by-item bar charts for individual workers

**Figure 3:**
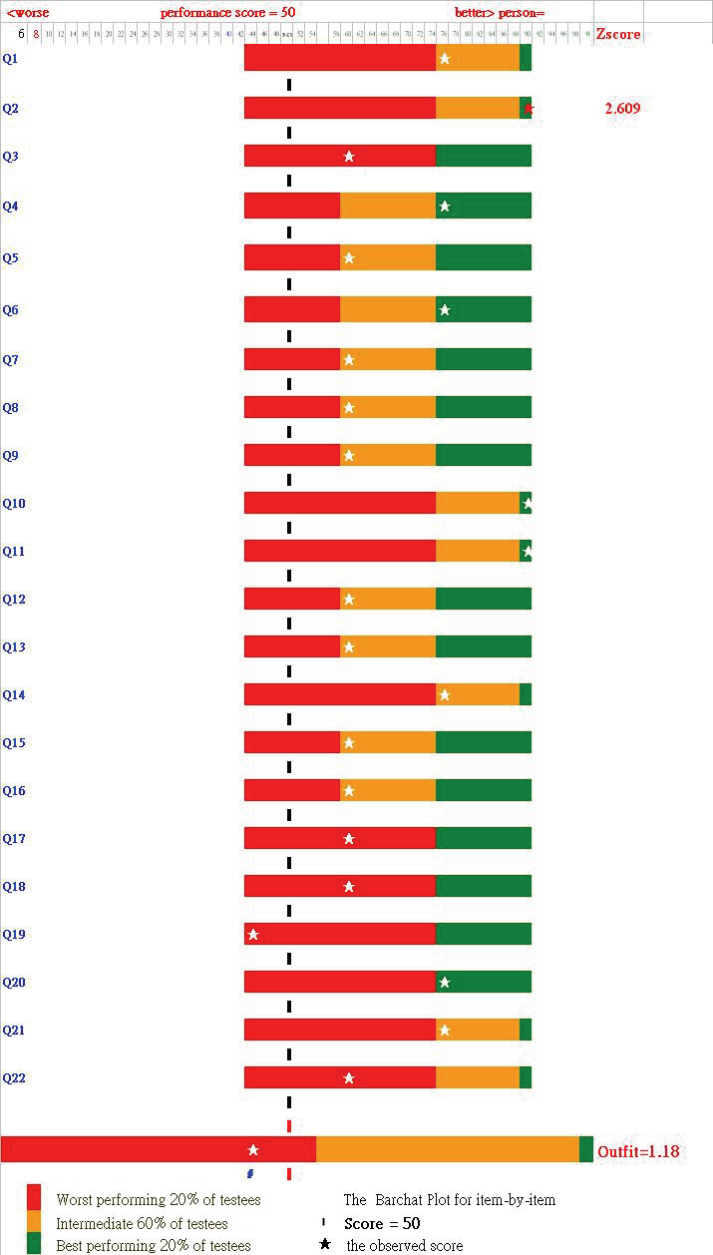
**Graphical representation of JCQ report in Excel showing examinee's aberrant response with item-by-item bar chart plot**.

Figure 3 shows item-by-item box plots for a particular worker. On the right-hand side of the figure, the outlier residual Z-score (2.609 on item 2) is shown, indicating that such items responding to this worker violating model's expectation are required for a further discussion, indicating that the observed score is greatly far apart from the expected response beyond the criterion of 1.96 (*p *< .05) when Rasch model is well fitted. Examining these residual Z-scores can help consultants focusing on a small number of key areas of concern for each worker in job strain.

### Graphical skewness analyses to screen out what groups we concern

**Figure 4:**
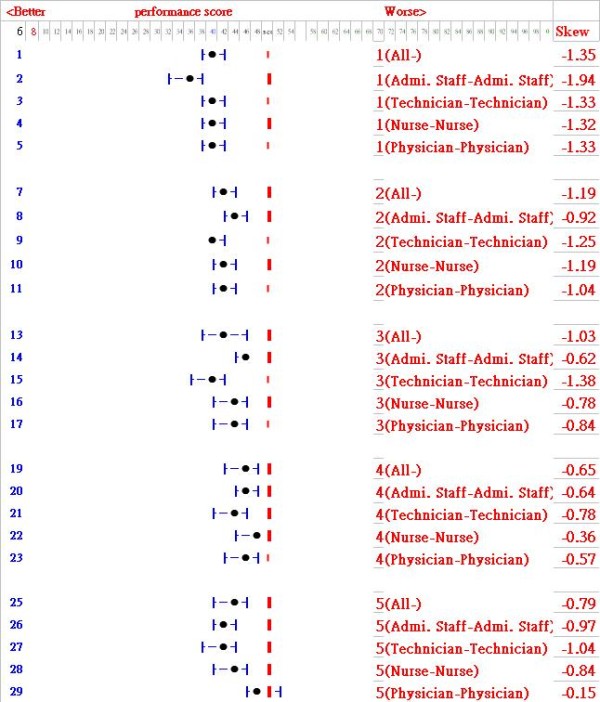
**Skewness analysis to detect extremely abnormal group**. Note: We are concerned with those groups with both positively skewed distribution.

If we are concerned with the occupational (or demographic) group which is with tremendous amount of job strains and stressors, those with positively skewed population would be selected. All of groups in the present study show less job stress with negatively skewed pattern in Figure 4. The skewness 95% CIs for each item showed can list such a juxtaposed comparison within (or between) group-by-group, item-by-item and year-by-year so as to see any difference or change occurred in sample dissimilarity of distribution in job stress suffered.

## Discussion

### Key findings

We found the prevalence rate of Chinese hospital workers with high job strain to be 16.5%. It is easy to make comparisons with the prevalence of job stress in other workplaces if the job strain derived from the C-JCQ is measured by Rasch analysis [29,30].

### What this adds to what was already known

We confirmed the C-JCQ with five dominant factors and ensured that Rasch analysis is an objective tool that can create linear and interval scores measuring job strain [31].

### What is the implication and what should be changed

Using Excel graphical representations of C-JCQ scores combined with WINSTEPS as a tool can help consultants focusing on a relatively small number of key areas of concern and allows for an easy, quick, and clear comparison of job strain, especially for those who are unfamiliar with a professional statistical package. Interested readers can download this feature in Additional files 1.

### The studied workplace verified as a symphony organization

The least satisfied scoring item was "I am free of conflicting demands that others make (shown in Table 2), indicating hospital employees commonly and frequently violate one of the Henri Fayol's 14 Principles of Management[32], Unity of Command (i.e., each worker should have only one boss with no other conflicting lines of command). Drucker stated that hospital organization is very similar to that of a music symphony orchestra (e.g., a conductor in orchestra like a surgeon in operation room not familiar with other instruments leads co-workers of a team to attain their goal) [33]. The most satisfied item was "My job requires that I learn new things" (shown in Table 2), indicating that the hospital is changing and improving frequently and the employees are satisfied with new thing to learn from their jobs.

### Strengths of the study

#### 1. Excel with Winsteps to implement Rasch analysis

The finding of Chinese hospital workers with a prevalence rate of high job strain at 16.5% can be compared with other studies based on the Job Strain Model paradigm. An Excel module (see addition file 1) combined with Winsteps as a tool which was never published before that so convenient and easy to use although Winsteps has been one of the most widely used programs for Rasch analysis in recent years [34]. It can do the following: 1) easily generate person measures with item anchored difficulties revealed in this study in comparison with other study using a C-JCQ scale; and 2) understandably depict C-JCQ results, showing the worker's strain level in a diagram that can compare studied samples scattered in the four quadrants (Figure 2) as well as item-by-item bar chart plots helping consultants focusing on job strain for each worker to be easily, quickly and clearly compared (Figure 3). Accordingly, the Excel module could be easier than that calculating a sum of weighted item scores for each subscale of those 22 items by the specific calculation formulas of the originators [1,12].

#### 2. Using skewness and its 95% CI to differentiate between groups on items

From management prospective, labor health protection and promotion have been recently emerged as an important issue [35,36]. Every workplace conducts enormous numbers of diverse mental examination for their employees. Using data to examinee differences of individual groups within or between groups, items and years becomes easy and possible if applying skewness coefficient and its 95% CI, which is shown in Figure 4.

#### 3. Allowing missing values in responses

Classic test theory (CTT) requires all items on a questionnaire to be completed in order to obtain a final score and evaluate the job stress resistant ability of the respondent. Rasch model is based on item response theory (IRT) and it allows missing data estimate examinee' stress resistant ability [37,38]. Readers who are interested can click it onto the website (http://www.webcitation.org/5akD0HfXm) with incompletely answered missing data to test the effects. The visual representation diagram on Internet for employee stress evaluation can be plotted. Individual job strain can be estimated even if missing data are in existence.

### Limitations and weaknesses of the study

High quality examinee feedback is important, so more work is needed to enable the administration of an effective examinee feedback tool in work site settings. In this study, we consider the questionnaire as a tool to collect individual's perception of job stress through a graphical representation. However, users may need some training to interpret four-quadrant diagram, item-by-item plots and skewness 95% CI comparison of job strain properly. The results imply that it is just useful for examining job stress in Chinese hospital work sites, thought the procedure and approach applied in this study can be repeated and carried out onto other cultures or onto other any population of people by promising researchers. The results regarding the job strain prevalence cannot be generalized to other workplaces in different cultures, although many translations of the JCQ translations have been used (http://www.jcqcenter.org/Translations.html).

The DL is constructed by two subscales of skill job discretion (6 items) and decision authority (3 items) although PA identified it composing two factors (correlation coefficient = 0.45). We averaged those two composed scores to yield the DL for plotting the four quadrant diagram (Figure 2) according to previous papers [1,12-14]. However, SD and DA are not conceptually the same construct so that they should not be used jointly to represent DL. It is worthy of additional study regarding JCQ to verify its proper application.

### Applications

The author-programmed Excel module is for application that is simple, understandable and easily used in the workplaces. All of the C-JCQ difficulties for items in each subscale have been anchored with WINSTEPS commands in the downloadable Excel module so that the results of job strain prevalence can be compared in the future. C-JCQ results for each worker can be reported in the four quadrants and item-by-item bar chart plots to show the job strain compared with the studied samples. Those two plots in Excel provide mental health consultants with information different from traditional approaches that cannot facilitate C-JCQ for emphasizing key areas of JCQ subscales to be easily, quickly and clearly compared and interpreted. Readers who are interested in assessing employees' job strain with the Rasch model can download it from Additional files 1 for practice. It must be noted that the Excel module must be placed in the same folder as the examples for WINSTEPS and the Excel application must be activated before activating the Excel module.

### Further Studies and Suggestions

Job strain conditions may be associated with the co-occurrence of adverse health behaviours that contribute to preventable chronic diseases [9]. Researchers and practitioners could put more emphases on the issues of self-evaluation for the public. The identification of occupational hazards that contribute to perceived workplace stress can better inform the development of interventions to reduce worker stress in acute care organizations in Taiwan. Future interventional research is needed to test interventions that are effective in the reduction of workplace stress related to occupational hazards, in order to promote improved workplace mental health. Research is needed to test interventions focused on 1) organizational climate change (i.e., changes in organizational structures and processes) for high risk groups and 2) improve worker's resistance to workplace stress to improve worker and patient outcomes.

## Conclusions

In practice, there may be more than one scaling questionnaire for job stress to be analyzed. We just studied the Job Content Questionnaire (JCQ) based on Karasek's Demand-Control-Support Model. In this study, IRT-based Rasch model was used to illustrate both four quadrants and item-by-item bar chart plots to examine worker's job strain. Future studies are encouraged to compare the prevalence rate of high job strain with other workplaces using this Excel module (in additional file 1). It is desirable to implement these procedures to evaluate data on both bases of individual and population. Future studies can carry out C-JCQ questionnaire with the Excel module with graphical representations to investigate workers' job strain in workplace settings.

## List of abbreviations

CTT: classic test theory; CS: coworker support; IRT: item response theory; DA: decision authority; DL: decision latitude; JCQ: job content questionnaire; PA: parallel analysis; PCA: principle component analysis; PD: psychological job demand; SD: skill discretion; SS: supervisor support; VBA: visual basic for application

## Competing interests

1. Authors' declaration of personal interests: None.

2. Declaration of funding interests: This study was funded in full by Chi-Mei Medical Center (Grant No. CMFHR 9820).

## Authors' contributions

TWC, WPL and SBS provided concepts and ideas for research design, writing, data analysis, facilities and equipment and fund procurement. SYH, HYW and SCC provided institutional liaison and project management. HRG and RVC provided consultation (including English revision and review of manuscript before submission) All authors read and approved the final manuscript.

## Pre-publication history

The pre-publication history for this paper can be accessed here:

http://www.biomedcentral.com/1471-2458/11/478/prepub

## Supplementary Material

Additional file 1 **Excel_VBA module for plotting graphical representations**. An Excel_VBA used to plot both four quadrant diagram and item-by-item bar chart plots. Click here for file
